# A Successful Switch From Ustekinumab to an Extended Dosing Interval of Guselkumab Without Induction in a Patient With Psoriasis Vulgaris

**DOI:** 10.7759/cureus.61567

**Published:** 2024-06-03

**Authors:** Kazuki Yatsuzuka, Jun Muto, Ken Shiraishi, Masamoto Murakami, Yasuhiro Fujisawa

**Affiliations:** 1 Department of Dermatology, Ehime University Graduate School of Medicine, Toon, JPN

**Keywords:** psoriasis vulgaris, ustekinumab, guselkumab, biologic treatment, psoriasis

## Abstract

Psoriasis vulgaris, also known as plaque-type psoriasis, is the most common form of psoriasis. It is characterized by erythematous plaques covered with scales. Among the available treatments, the fully human monoclonal antibodies ustekinumab (UST) and guselkumab (GUS) have low immunogenicity. Additionally, GUS has not been found to have a significant risk of inducing the development of clinically relevant neutralizing antibodies. Therefore, we sometimes consider switching to GUS when UST is insufficiently effective. However, switching to another biological agent usually requires an induction phase, potentially incurring additional costs. We herein present the first case of a successful transition from UST 90 mg to an extended dosing interval of GUS without an induction phase. This approach may be a viable and cost-saving option, especially for patients with relatively low disease activity.

## Introduction

Psoriasis vulgaris (PsV) is a chronic inflammatory skin disorder characterized by erythematous plaques covered with silver or gray scales. It affects approximately 2% to 3% of the global population and significantly impacts quality of life [1]. Many treatment options are available, including topical steroids or vitamin D, ultraviolet light therapy, vitamin A derivatives, and immunosuppressants. The most advanced treatments developed in the past decade are biological agents. Four interleukin-12/23 and interleukin-23 inhibitors are available. Among them, ustekinumab (UST) and guselkumab (GUS) are fully human monoclonal antibodies with low immunogenicity. Indeed, GUS has not been found to have a significant risk of inducing the development of clinically relevant neutralizing antibodies [2]. Although UST has been widely used, some patients may exhibit resistance or suboptimal responses. In such cases, switching to an alternative biologic agent becomes necessary. It may seem reasonable to consider switching to GUS when a patient exhibits resistance to UST. However, UST is administered every 12 weeks (Q12W), whereas GUS typically requires dosing every eight weeks (Q8W). Additionally, switching to another biologic agent usually requires an induction phase, potentially incurring additional costs. To the best of our knowledge, this is the first case of a PsV patient who successfully transitioned from UST 90 mg to GUS, adopting an extended dosing interval (Q12W) without undergoing an induction phase.

## Case presentation

**Figure 1:**
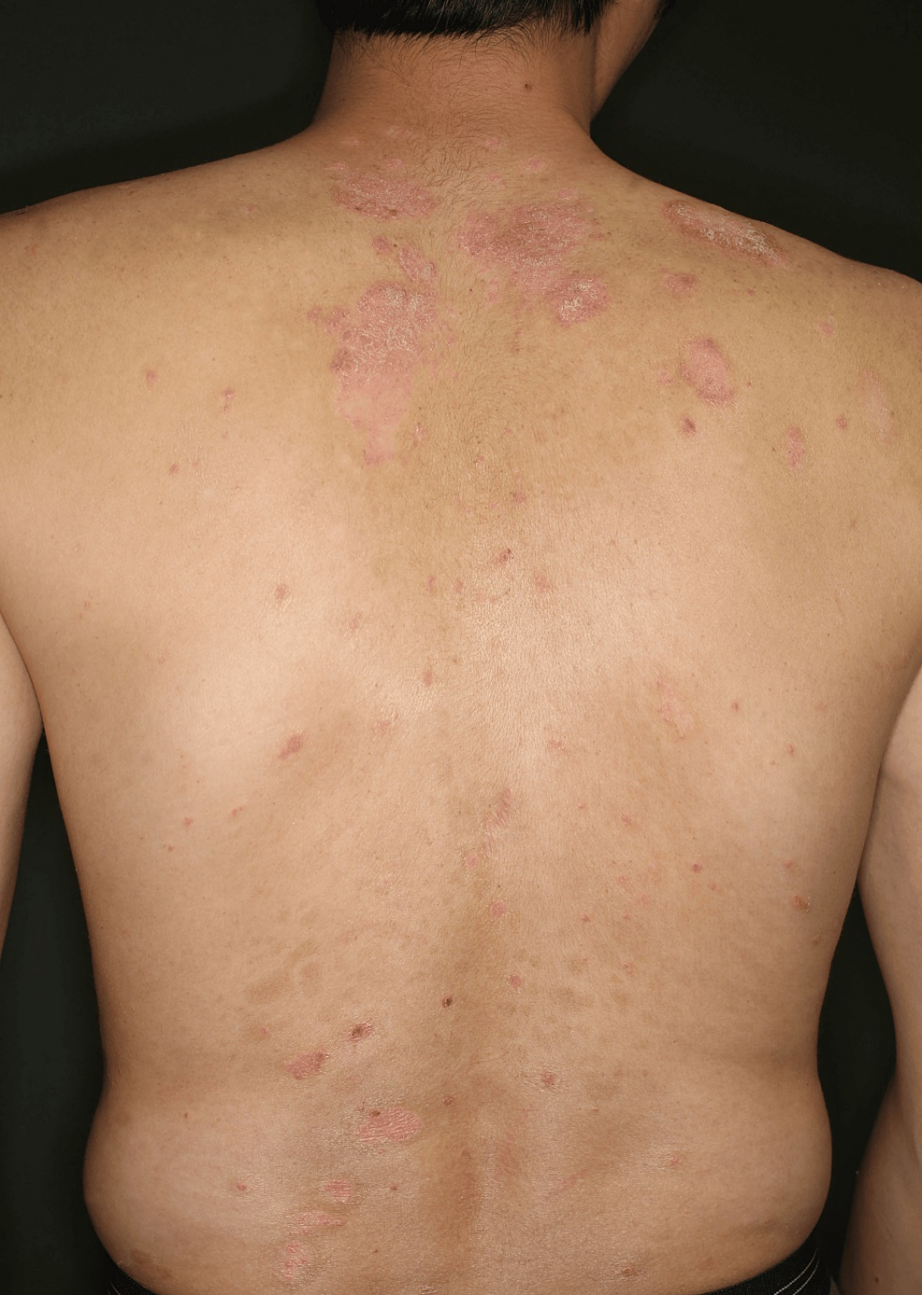
Patient’s image before starting biologics Prior to UST treatment, scaly erythematous plaques were evident on the scalp, extremities (no photograph), and trunk. UST, ustekinumab

A 33-year-old man with a 13-year history of PsV came to our department in 2015, displaying scaly and erythematous plaques on his scalp, trunk, and extremities. The skin lesions were unresponsive to topical therapies. Due to his refusal to use an autoinjector, we prescribed UST 45 mg at weeks 0 and 4 and then Q12W, which improved his Psoriasis Area and Severity Index (PASI) score from 12.1 (Figure 1) to 2.3.

**Figure 2:**
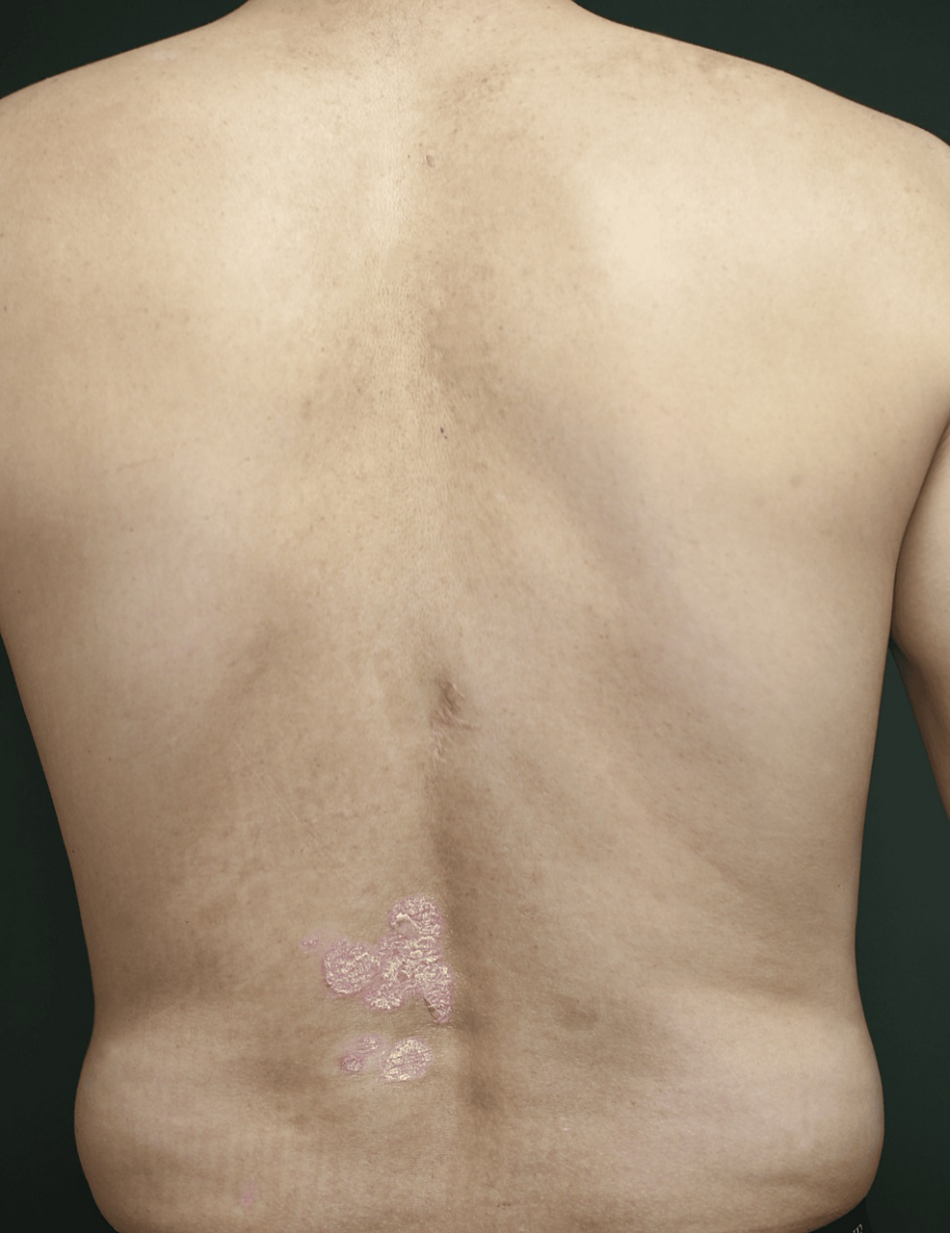
Patient’s image before switching from UST to GUS Refractory skin lesions were present on the lower back before transitioning to GUS. GUS, guselkumab; UST, ustekinumab

With unsatisfactory improvement on the scalp, back, and lower extremities, we increased the dose to 90 mg in 2021. However, this adjustment failed to yield better results, and by 2023, his PASI score had risen to 3.7 (Figure 2).

**Figure 3:**
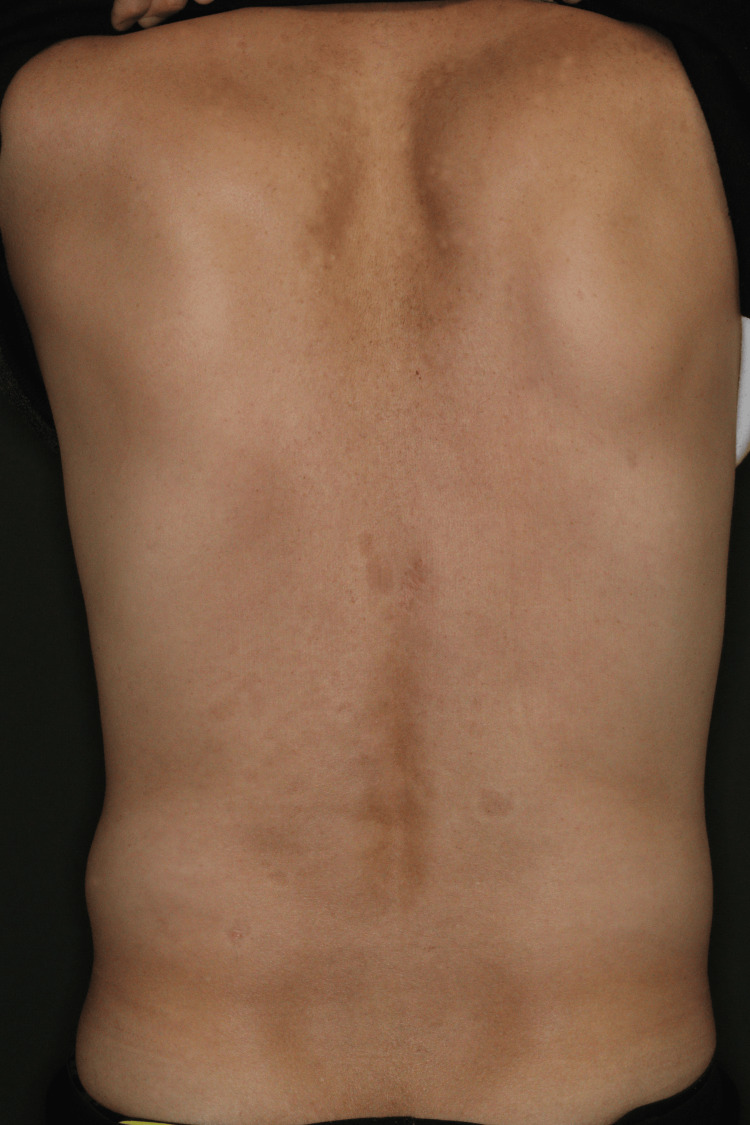
Patient’s image after switching from UST to GUS The lesions resolved nine months after the switch. GUS, guselkumab; UST, ustekinumab

Despite his strong preference for switching to another fully human monoclonal antibody, he wished to avoid both an induction phase and a reduced dosing interval due to logistical and financial constraints. With the patient’s agreement, we transitioned from UST 90 mg to GUS 100 mg Q12W without induction. After nine months, his PASI score decreased to 0.3 (Figure 3), with minimal erythema remaining on the scalp.

## Discussion

The development of systemic treatments for PsV has been remarkable in recent years. Oral systemic treatments include acitretin, etretinate, fumarates, cyclosporin, methotrexate, apremilast, deucravacitinib, and Janus kinase inhibitors. Biological agents include tumor necrosis factor inhibitors (infliximab, adalimumab, etanercept, and certolizumab pegol), interleukin-12/23 inhibitors (UST), interleukin-17 inhibitors (secukinumab, ixekizumab, brodalumab, and bimekizumab), and interleukin-23 inhibitors (GUS, risankizumab, and tildrakizumab). Despite this progress, a Japanese guideline regarding the treatment of PsV patients has not emerged, whereas several global guidelines are available [3-5]. Among these, according to a systematic review [6], the EuroGuiDerm guideline concerning the systemic treatment of PsV is the only clinical practice guideline to have achieved excellent quality across all six Appraisal of Guidelines for Research and Evaluation II domains, to not raise Lenzer’s red flags, and to have higher trustworthiness according to the US Institute of Medicine’s criteria. However, even this guideline fails to provide specific details, such as which biological agent should be used first and in what order the changes should be made. For example, which of the interleukin-23 inhibitors should be used first? Therefore, the accumulation of real-world usage reports, such as our report, is essential to build evidence.

Four interleukin-12/23 and interleukin-23 inhibitors are currently available. UST and GUS are fully human monoclonal antibodies with low immunogenicity. Indeed, no significant risk of inducing the development of clinically relevant anti-drug antibodies has been identified with GUS. Antibodies to biological agents have been described in relation to serious adverse events, such as thromboembolic events, lupus-like syndrome, vasculitis-like events, and other autoimmune manifestations mediated by immune complexes [7]. Thus, from an immunogenicity perspective, a switch to GUS (rather than the humanized monoclonal antibodies risankizumab and tildrakizumab) may be preferable in patients with resistance to UST.

The high cost associated with biologics for PsV leads some patients to discontinue treatment for financial reasons. Some patients wish to maintain the same dosing interval when transitioning between biologics. Traditionally, switching from UST to GUS involves shortening the dosing interval and includes an induction phase [8]. However, studies from Europe have explored optimizing the GUS dosing interval post-UST transition [9,10]. A retrospective study reported that 10 psoriasis patients experienced satisfactory outcomes by switching to GUS Q12W without induction, in comparison to GUS Q8W with induction [10]. Notably, the mean baseline PASI score at the time of switching was 3.55, considerably lower than that of the standard care group (14.53) [10]. Furthermore, a phase 2 trial of GUS indicated that a dose of GUS 50 mg Q12W was effective even with a high baseline PASI score [11]. Transitioning from UST to GUS Q12W without induction may be a viable option, particularly for patients with relatively low disease activity, as in our case. However, it has been reported that anti-drug antibodies are more likely to appear when blood drug concentrations decrease due to prolonged dosing intervals [7]. We need to consider this risk and be cautious when optimizing the dosing interval.

## Conclusions

This case highlights the feasibility of transitioning from UST to GUS without an induction phase, particularly for patients with relatively low disease activity. To the best of our knowledge, this is the first report of a patient with PsV who has successfully transitioned from UST 90 mg to GUS Q12W without induction. Individualized treatment strategies in terms of patient preferences and logistical factors play a crucial role in optimizing outcomes. Further clinical studies are warranted to validate this approach and explore its potential impact on medical costs.

## References

[1] Damiani G, Bragazzi NL, Karimkhani Aksut C (2021). The global, regional, and national burden of psoriasis: results and insights from the Global Burden of Disease 2019 study. Front Med (Lausanne).

[2] Tsakok T, Rispens T, Spuls P, Nast A, Smith C, Reich K (2021). Immunogenicity of biologic therapies in psoriasis: myths, facts and a suggested approach. J Eur Acad Dermatol Venereol.

[3] Smith CH, Yiu ZZ, Bale T (2024). British Association of Dermatologists guidelines for biologic therapy for psoriasis 2023: a pragmatic update. Br J Dermatol.

[4] Nast A, Smith C, Spuls PI (2020). EuroGuiDerm Guideline on the systemic treatment of psoriasis vulgaris - part 1: treatment and monitoring recommendations. J Eur Acad Dermatol Venereol.

[5] Nast A, Smith C, Spuls PI (2021). EuroGuiDerm Guideline on the systemic treatment of psoriasis vulgaris - part 2: specific clinical and comorbid situations. J Eur Acad Dermatol Venereol.

[6] Yen H, Huang CH, Huang IH (2022). Systematic review and critical appraisal of psoriasis clinical practice guidelines: a Global Guidelines in Dermatology Mapping Project (GUIDEMAP). Br J Dermatol.

[7] Jani M, Dixon WG, Chinoy H (2018). Drug safety and immunogenicity of tumour necrosis factor inhibitors: the story so far. Rheumatology (Oxford).

[8] Langley RG, Tsai TF, Flavin S (2018). Efficacy and safety of guselkumab in patients with psoriasis who have an inadequate response to ustekinumab: results of the randomized, double-blind, phase III NAVIGATE trial. Br J Dermatol.

[9] Berenguer-Ruiz S, Rivera R, Herranz P (2022). Ustekinumab to guselkumab transitions: a series of 54 patients emulating the navigate trial in real life. Dermatol Ther.

[10] Ruiz-Villaverde R, Chinchay FV, Rodriguez-Fernandez-Freire L, Armario-Hita JC, Pérez-Gil A, Galán-Gutiérrez M (2022). Guselkumab dosing interval optimization in adult patients with moderate-to-severe psoriasis switching from ustekinumab. Dermatol Ther.

[11] Gordon KB, Duffin KC, Bissonnette R (2015). A phase 2 trial of guselkumab versus adalimumab for plaque psoriasis. N Engl J Med.

